# Cell cycle-dependent inhibition of 53BP1 signaling by BRCA1

**DOI:** 10.1038/celldisc.2015.19

**Published:** 2015-08-04

**Authors:** Lin Feng, Nan Li, Yujing Li, Jiadong Wang, Min Gao, Wenqi Wang, Junjie Chen

**Affiliations:** 1 Department of Experimental Radiation Oncology, The University of Texas MD Anderson Cancer Center, Houston, TX, USA; 2 State Key Laboratory of Oncology in South China, Collaborative Innovation Center for Cancer Medicine, Sun Yat-Sen University Cancer Center, Guangzhou, China; 3 State Key Laboratory for Biocontrol and Key Laboratory of Gene Engineering of Ministry of Education, School of Life Sciences, Sun Yat-sen University, Guangzhou, China; 4 Institute of Systems Biomedicine, Medical Isotopes Research Center, School of Basic Medical Sciences, Peking University, Beijing, China

**Keywords:** BRCA1, 53BP1, PTIP, RIF1, ATM, cell cycle, homologous recombination, DNA repair choice

## Abstract

DNA damage response mediator protein 53BP1 is a key regulator of non-homologous end-joining (NHEJ) repair. 53BP1 protects DNA broken ends from resection by recruiting two downstream factors, RIF1 (RAP1-interacting factor 1) and PTIP (Pax transactivation domain-interacting protein), to double-stranded breaks (DSBs) via ATM (ataxia telangiectasia mutated)-mediated 53BP1 phosphorylation, and competes with BRCA1-mediated homologous recombination (HR) repair in G1 phase. In contrast, BRCA1 antagonizes 53BP1-direct NHEJ repair in S/G2 phases. We and others have found that BRCA1 prevents the translocation of RIF1 to DSBs in S/G2 phases; however, the underlying mechanism remains unclear. Here we show that efficient ATM-dependent 53BP1 phosphorylation is restricted to the G1 phase of the cell cycle, as a consequence RIF1 and PTIP accumulation at DSB sites only occur in G1 phase. Mechanistically, both BRCT and RING domains of BRCA1 are required for the inhibition of 53BP1 phosphorylation in S and G2 phases. Thus, our findings reveal how BRCA1 antagonizes 53BP1 signaling to ensure that HR repair is the dominant repair pathway in S/G2 phases.

## Introduction

DNA double-stranded breaks (DSBs) arise from many sources, including exposure to ionizing radiation, reactive oxygen species and collapse of replication forks. DSBs must be repaired properly to ensure genomic stability. There are two major pathways for repairing DSBs: homologous recombination (HR) and non-homologous end joining (NHEJ). DNA repair mechanisms differ at different stages of the cell cycle. In S and G2 phases, cells use HR repair to rejoin DNA breaks when homolog sequences on the sister chromatid are present. In G1 phase, as the sister chromatid is absent, NHEJ is the major pathway for DSB repair. The choice of DSB repair by HR or NHEJ is also dictated by the 5ʹ–3ʹ DNA end resection at break sites; extensive end degradation is an essential step in HR, whereas NHEJ can only occur at DNA ends with limited or no processing.

BRCA1 (breast cancer 1, early onset) is a well-known tumor suppressor that has a critical role in DNA repair. BRCA1 harbors a highly conserved amino terminal RING domain and tandem BRCT domains at its carboxyl terminus. BRCT domains are phosphopeptide recognition modules that participate in the assembly of protein complexes required for DNA repair functions. The RING domain of BRCA1 confers E3 ubiquitin ligase activity to BRCA1 via its ability to interact with ubiquitin-conjugating enzymes (E2) and BARD1 [1]. The major cause of the genetic instability associated with BRCA1 deficiency is the impaired ability of *BRCA1* mutant cells to undergo HR-mediated DSB repair. This inability forces cells to rely on alternative non-template-based repair pathways, such as NHEJ, which could result in chromosome aberrations and/or translocation. BRCA1 seems to act at multiple stages during HR, as BRCA1 facilitates both early (DNA end resection and single-stranded DNA (ssDNA) formation) and late (RAD51-mediated DNA strand exchange) phases of HR.

At present, the precise mechanisms by which BRCA1 functions in HR remain unclear, but the antagonistic relationship between BRCA1 and another DNA repair protein, 53BP1 (p53- binding protein 1), during DNA repair pathway choice have been well established in recent years. 53BP1 is a key positive regulator of NHEJ-mediated DSB repair and protects broken DNA ends from processing, a process that is promoted by BRCA1 [2, 3]. Both the physical presence of 53BP1 at DSBs and the phosphorylation of 53BP1 by ATM (ataxia telangiectasia mutated) kinase are required for the anti-HR activities of 53BP1 [4]. We and other researchers have found that ATM-phosphorylated 53BP1 recruits a mediator protein, RIF1 (RAP1-interacting factor 1) to DNA damage sites to protect broken DNA ends from BRCA1-mediated end resection and subsequent HR repair [5–8]. PTIP (Pax transactivation domain-interacting protein) was discovered as another phospho-53BP1 binding protein that is also required for 53BP1-mediated inhibition of BRCA1-directed HR and the sensitivity to poly ADP ribose polymerase (PARP) inhibitor in BRCA1 mutation cancer cells [9]. Therefore, 53BP1 acts as a scaffold protein to recruit two downstream effectors, RIF1 and PTIP, at DSBs to inhibit HR and directs DNA repair toward NHEJ. On the other hand, 53BP1’s NHEJ-promoting function is also inhibited by BRCA1, as loss of BRCA1 enables the accumulation of RIF1 at DSBs in S/G2 phase cells and inhibits initial resection of DNA ends [5, 6, 10].

The available evidence clearly shows that the balance of BRCA1 and 53BP1 controls the choice of DNA repair pathways, and that this balance is cell cycle regulated. However, essentially nothing is known about the mechanism by which BRCA1 suppresses 53BP1 pathway in S/G2 phases. Here, using synchronized cells, we found that BRCA1 inhibits the phosphorylation of 53BP1 by ATM and therefore prevents the subsequent accumulation of RIF1 and PTIP at DSB sites in S/G2 phases of cell cycle. Thus, the 53BP1-initiated NHEJ repair is confined in G1 phase.

## Results

### Opposite regulations of DSB-induced foci formation between PTIP and BRCA1

**Figure 1:**
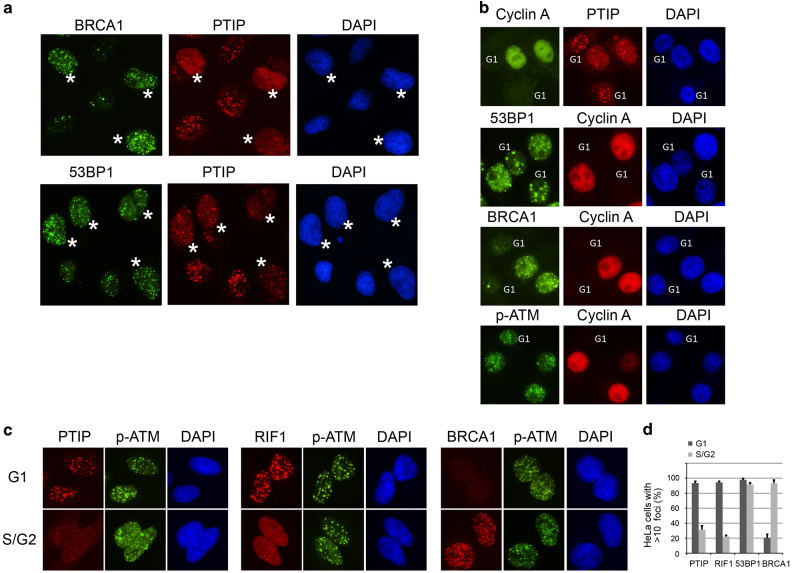
Cell cycle-dependent PTIP (Pax transactivation domain-interacting protein) foci formation. (**a**) Asynchronous HeLa cells were treated with γ-irradiation (ionizing radiation (IR), 10 Gy) and recovered for 4 h before fixation and stained with antibodies as indicated. Asterisks indicate PTIP foci-negative cells. (**b**) Asynchronous HeLa cells were treated as in **a** and co-stained with S phase marker, cyclin A. G1 phase cells were indicated. (**c**) Representative images showing PTIP, RIF1 (RAP1-interacting factor 1) and BRCA1 foci. HeLa cells were enriched in G1 phase by treating with nocodazole and then released for 6 h. S/G2 phase cells were enriched by double-thymidine block and released for 6 h. Two  hours before fixation, cells were irradiated with 10 Gy of IR. (**d**) A histogram showing the percentage of cells containing >10 nuclear foci. Experiments were performed in triplicate and at least 100 cells were counted in each experiment for each treatment.

We and others have demonstrated that BRCA1 and RIF1 display mutually exclusive foci formation in response to ionizing radiation (IR) [5, 6, 11]. Because the accumulation of PTIP, another 53BP1 downstream effector, at DSB sites is also ATM- and 53BP1-dependent but RIF1-independent [9], we decided to investigate the potential cell cycle-dependent regulation of PTIP localization at DSBs. We generated a PTIP polyclonal antibody that was highly specific for western blotting and immunofluorescence staining. The polyclonal antibody only recognized a band of ~130 kDa (PTIP-predicated molecular weight: 121 kDa) and the band was undetectable in PTIP knockout cells generated using CRISPR/Cas9 system (Supplementary Figure S1a). Using this antibody for immunostaining, we found that PTIP is a pan-nuclear protein in undamaged HeLa cells and form foci after exposure to γ-irradiation. In addition, the foci formation of PTIP were 53BP1 and ATM dependent (Supplementary Figure S1b), as previously reported [9]. Strikingly, although 53BP1 foci formed in over 90% of the dividing cells, only ~60% of cells showed discernible PTIP foci formation (Figure 1a). When we co-stained these cells with BRCA1 and PTIP antibodies, we found that PTIP and BRCA1 showed mutually exclusive foci formation (Figure 1a). Cells co-stained with S and G2 cell cycle marker cyclin A further supported that PTIP foci were absent in S/G2 cells, and this phenomenon was also observed in other cell lines including non-tumorigenic breast epithelial cell line MCF10A (Supplementary Figure S1c). On the contrary, 53BP1 foci formation and ATM activation occurred in all phases of the cell cycle, whereas BRCA1 only translocated to DSBs in the S/G2 phases of cells (Figure 1b), as previously reported [5]. We also utilized laser-induced microirradiation to generate DSBs, and similar results were obtained. PTIP failed to be recruited to laser-induced DNA damage sites where strong BRCA1 strips formed and vice versa (Supplementary Figure S2). To further elucidate the cell cycle dependency of PTIP foci formation, we synchronized the cells in G1 or S/G2 phases and then treated with irradiation. The results showed that, as with RIF1, PTIP only translocates to DSB sites in G1 phase, but not in S/G2 phases, which is opposite to that of BRCA1 (Figures 1c and d). The diminished foci formation of PTIP and RIF1 in the S/G2 phases was not attributable to defective ATM activation, as ATM autophosphorylation on Ser1981 occurred in all stages of the cell cycle (Figures 1b and c). Together, these data indicate that the accumulation of PTIP at DSB sites is similar to RIF1, which only occurs in G1 cells.

### ATM-dependent 53BP1 phosphorylation is G1 phase specific

**Figure 2:**
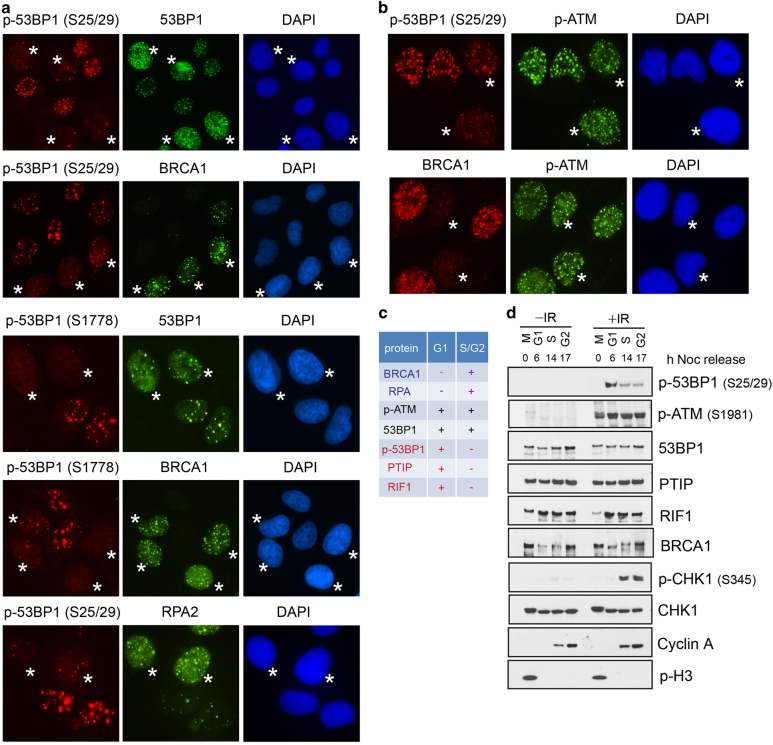
Cell cycle-dependent 53BP1 phosphorylation by ATM (ataxia telangiectasia mutated). (**a**) Asynchronous HeLa cells were treated as described in Figure 1a, arrows indicate p-53BP1 foci-negative cells. Cells with >10 foci following ionizing radiation (IR) were counted as positive cells. (**b**) HeLa cells were processed as in **a**, and the asterisks indicate BRCA1 or p-53BP1 foci-negative cells. The percentage of asynchronous cells forming indicated foci were as follows: S25/S29 (58%), S1778 (60%), 53BP1 (98%), BRCA1 (65%), RPA2 (52%) and p-ATM (94%), at least 200 cells were counted. (**c**) Summary of cell cycle-dependent foci formation of BRCA1 and 53BP1 complexes. (**d**) HeLa cells were arrested in M phase with nocodazole (100 ng ml^−1^) treatment for 16 h and then released for different time periods. The cells were irradiated with 10 Gy of IR 1 h or left untreated before harvesting.

As PTIP and RIF1 share the same mechanism for their translocation to DSBs (that is, binding to ATM-phosphorylated 53BP1), we next asked whether the level of 53BP1 phosphorylation would change in different phases of cell cycle in response to IR. We used two commercially available antibodies that recognize ATM phosphorylation sites on 53BP1: anti-S25/29 and anti-S1778 antibodies. These two phospho-antibodies specifically recognize the phosphorylated forms of 53BP1 because the stainings were induced by IR and were undetectable in 53BP1 knockout cells generated using CRISPR/Cas9 technology (Supplementary Figure S3a). In addition, phosphorylation on these S/TQ sites were ATM dependent, as ATM inhibitor KU55933, but not DNA-PK inhibitor NU7026, efficiently abolished IR-induced 53BP1 phosphorylation at S25/29 and S1778 sites (Supplementary Figure S3b). Immunostaining with these two phospho-53BP1 antibodies revealed that not all 53BP1 foci-positive cells exhibited positive 53BP1 phosphorylation after irradiation, and the signal of 53BP1 phosphorylation was greatly diminished in BRCA1-positive cells (that is, cells in the S/G2 phases). Similarly, IR-induced replication protein A (RPA) foci formed only in cells that did not exhibit p-53BP1 staining (Figure 2a). These data suggest that DNA damage-induced 53BP1 phosphorylation mainly occurs in G1 cells. The G1-specific phosphorylation of 53BP1 by ATM is unique because ATM autophosphorylation on Ser1981 occurred in all phases of the cell cycle regardless of BRCA1 status (Figures 1b and c, and 2b). Moreover, we tested other ATM substrates. Immunostaining with pan-ATM substrates antibody, which recognizes the S*/T*Q motif [12], also suggested that the phosphorylation of ATM substrates occur in all cell cycle phases (Supplementary Figure S4). Similarly, γH2AX and KAP-1 were phosphorylated throughout the cell cycle (Supplementary Figure S4). However, phosphorylation of NBS1 coincided with BRCA1 foci formation (Supplementary Figure S4), indicating that NBS1 may be phosphorylated by both ATM and Ataxia telangiectasia and Rad3-related protein (ATR) in S/G2 cells. The cell cycle-dependent foci formation of these DNA repair proteins are summarized in Figure 2c.

To confirm these results and ensure that the inverse correlation between phospho-53BP1 and BRCA1 was not resulting from cell cycle-dependent changes in protein levels, we examined IR-induced 53BP1 phosphorylation in synchronized cells. HeLa cells were arrested in mitosis by nocodazole treatment, released into G1, S and G2 phases, and were irradiated or left untreated. 53BP1 phosphorylation peaked in G1 phase and then quickly decreased after cells entered S and G2 phases—as judged by cyclin A levels. Consistently, ATM was activated regardless of cell cycle stages. The expression of 53BP1 complex (that is, 53BP1, PTIP and RIF1) did not change appreciably throughout the cell cycle, only RIF1 showed a reduction in mitosis. BRCA1 level was low in G1 phase, increased when cells enter S phase and reached the maximum in G2 phase, and IR treatment induced phosphorylation of BRCA1 during S and G2 phases, as indicated by mobility shift. In contrast to 53BP1 phosphorylation, CHK1 phosphorylation was only observed in S and G2 cells (Figure 2d), which was consistent with the report that ATR activation is restricted to S/G2 phases [13]. Taking together, our data indicate that effective ATM-induced 53BP1 phosphorylation is limited to G1 phase.

### Recovered 53BP1 phosphorylation, PTIP and RIF1 foci formation in S/G2 cells by BRCA1 deficiency

**Figure 3:**
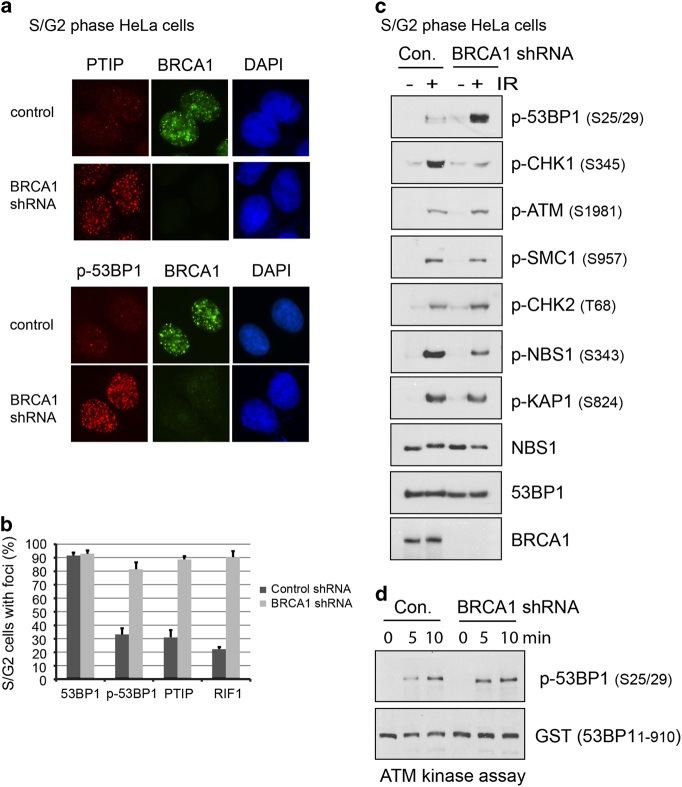
BRCA1 inhibits 53BP1 phosphorylation. (**a**) BRCA1 deficiency restored PTIP (Pax transactivation domain-interacting protein) and p-53BP1 foci formation in S/G2 phase cells. HeLa cells infected with lentivirus carrying non-target or BRCA1 short hairpin RNAs were synchronized in S/G2 and irradiated as described in Figure 1c. (**b**) Quantification of 53BP1 complex foci formation, the data are represented as the mean±s.e. (*n*=3). More than 100 cells were counted to determine the percentages of foci forming cells in each sample. (**c**) Depletion of BRCA1 increases 53BP1 phosphorylation by ATM (ataxia telangiectasia mutated). Control and BRCA1 knockdown cells were synchronized in S/G2 phases as described in Figure 1c. Cells were mocked-treated or treated with ionizing radiation. One hour after ionizing radiation, cells were harvested, and the cell lysates were analyzed by immunoblotting using the indicated antibodies. (**d**) ATM kinase assays were performed with anti-ATM immunoprecipitates prepared from control (Con.) or BRCA1-depleted cells. ATM kinase was incubated with the purified GST-53BP1 fusion protein (1–910 aa) for the times as indicated. The phosphospecific antibody against phospho-Ser25 and Ser29 of 53BP1 was used for western blotting to detect phosphorylated 53BP1. IR, ionizing radiation; shRNA, short hairpin RNA.

Loss of BRCA1 restored RIF1 re-localization to DSBs in S/G2 phase cells [5, 6, 10]. As the cell cycle-specific foci formation of PTIP and p-53BP1 resembled that of RIF1 (Figures 1 and 2), we investigated whether DSBs accumulation of PTIP and p-53BP1 were also affected by the BRCA1 status. HeLa cells infected with control or BRCA1 short hairpin RNAs were synchronized in the S/G2 phases by double-thymidine block. Immunofluorescence data showed that ablation of BRCA1 fully restored PTIP and p-53BP1 accumulation at DSBs (Figure 3a and b). Western blot results also indicated that BRCA1 depletion increased 53BP1 phosphorylation levels in S/G2 cells. As expected, CHK1 phosphorylation, an indicator for DNA end resection and ssDNA formation [14], was greatly reduced by BRCA1 deficiency. Consistent with immunostaining results, ATM autophosphorylation was not affected by BRCA1 loss.

We also examined other known ATM substrates such as SMC1, CHK2, KAP-1 and NBS1. The phosphorylation levels of SMC1, CHK2 and KAP-1 were not affected by BRCA1 deficiency. The only exception was NBS1, phosphorylation of which is S/G2 specific (Supplementary Figure S4) and declined in the absence of BRCA1 (Figure 3c). The specific mechanism by which BRCA1 stimulates NBS1 phosphorylation is currently unknown, but the cell cycle-dependent complex formation of BRCA1 and NBS1 might contribute to this regulation [15]. An alternative explanation for the regulation could be that NBS1 is also a substrate of ATR [16], which is activated following IR in a BRCA1-dependent manner [17].

To further exclude the possibility that BRCA1 directly regulates ATM kinase activity, we generated GST-53BP1 fusion protein (1–910 aa) that contains multiple ATM phosphorylation sites (S/TQ sites) as substrates and co-incubated with endogenous ATM kinase that was immunoprecipitated from normal or BRCA1-deficient cells. The *in vitro* kinase assay results suggest that ATM immunoprecipitates prepared from control or BRCA1-deficient cells were capable of phosphorylating recombinant 53BP1 to similar extent (Figure 3d). Thus, BRCA1 likely inhibits 53BP1 phosphorylation *in vivo* without affecting ATM activation and/or ATM kinase activity.

We next investigated whether the restored 53BP1 phosphorylation and PTIP foci formation are direct results of BRCA1 deficiency or are indirectly mediated by DNA end resection defect accompanied with BRCA1 loss. Because CtBP-interacting protein (CtIP) is a BRCA1-associated protein that participates in DNA end processing [18, 19], we depleted CtIP and synchronized the cells in the S/G2 phases. Immunostaining data suggested that CtIP has only a minimal role in antagonizing 53BP1 signaling because few PTIP and p-53BP1 foci formation were observed in S/G2 phases of cells lacking CtIP (Supplementary Figure S5a); however, severe resection defects occurred in both CtIP- and BRCA1-depleted cells (Supplementary Figure S5b). These observations are consistent with our previous results on RIF1 [5]. Therefore, hyper-activation of DNA end resection machinery in S and G2 phases does not in itself cause a defect in 53BP1 signaling.

### Both BRCT and RING domains of BRCA1 are required for 53BP1 inhibition

**Figure 4:**
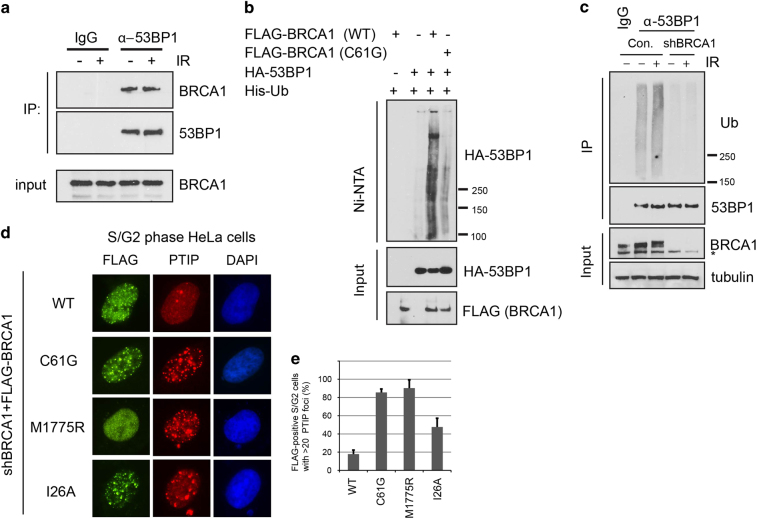
The E3 ubiquitin ligase activity of BRCA1 is required for inhibition of 53BP1 signaling. (**a**) 293T cells were untreated or irradiated at 10 Gy and processed 1h later. Cell lysates were prepared and immunoprecipitated (IP) using rabbit immunoglobulin G (IgG) or anti-53BP1 antibody. (**b**) BRCA1 ubiquitinates 53BP1 *in vivo*. 293T cells were transfected with His-ubiquitin (Ub), SFB-BRCA1/BARD1 and HA-53BP1. The pull-down and immunoblotting analysis was conducted as indicated. (**c**) HeLa cells infected with lentivirus carrying control or BRCA1 short hairpin RNAs were irradiated or untreated. Total cell lysates were prepared. Immunoprecipitation using high-salt buffer was performed with normal rabbit IgG or anti-53BP1 antibody. Endogenous 53BP1 ubiquitination was detected with anti-Ub antibody (see Materials and Methods for details). (**d**) BRCA1-depleted HeLa cells were transfected with various BRCA1 constructs, synchronized in S/G2 phases and processed as described in Figure 1c. (**e**) Quantification of PTIP (Pax transactivation domain-interacting protein) foci formation by the indicated BRCA1 constructs, as described in **c**. More than 30 FLAG-positive cells and the cells with >20 PTIP foci were counted, results are presented as the means (±s.d.) of three independent experiments. Ni-NTA, nickel-nitrilotriacetic acid; WT, wild type.

Next, we investigated the mechanism by which BRCA1 inhibits 53BP1 phosphorylation. Mass spectrometry analysis results of tandem affinity purification of BRCA1 and BARD1 complexes identified 53BP1 as a BRCA1-binding protein (Supplementary Figure S6a). Reciprocal co-immunoprecipitation experiments of endogenous BRCA1 and 53BP1 also confirmed the physical interaction between BRCA1 and 53BP1; however, the binding was not affected by IR (Figure 4a). Because BRCA1 and 53BP1 participate in opposite repair pathways and the only known enzymatic function of BRCA1 is its E3 ubiquitin ligase activity, which is mediated by the N-terminal RING domain of BRCA1, we tested whether BRCA1 could ubiquitinate 53BP1 *in vivo*. Co-overexpression of 53BP1 with wild-type BRCA1 induced robust ubiquitination of 53BP1; however, co-transfection with a BRCA1 C61G mutant, which included a mutation in the RING domain targeting the critical Zn^2+^-chelating residue, resulted in reduction of 53BP1 ubiquitination (Figure 4b). Furthermore, the ubiquitination of endogenous 53BP1 was greatly reduced in cells depleted of BRCA1, while IR treatment led to slightly increased 53BP1 ubiquitination (Figure 4c). We searched for the regions on 53BP1 that could be ubiquitinated by BRCA1. *In vitro* ubiquitination assays revealed that several regions at the C terminus of 53BP1 could be ubiquitinated by BRCA1/BARD1 complex (Supplementary Figure S6b), indicating that BRCA1 may ubiquitinate multiple residues on the C terminus of 53BP1. However, so far, we could not identify the BRCA1-dependent ubiquitination sites on 53BP1. Thus, we could not conclude whether or not BRCA1-dependent ubiquitination of 53BP1 has any role in BRCA1-mediated suppression of 53BP1 phosphorylation and function.

**Figure 5:**
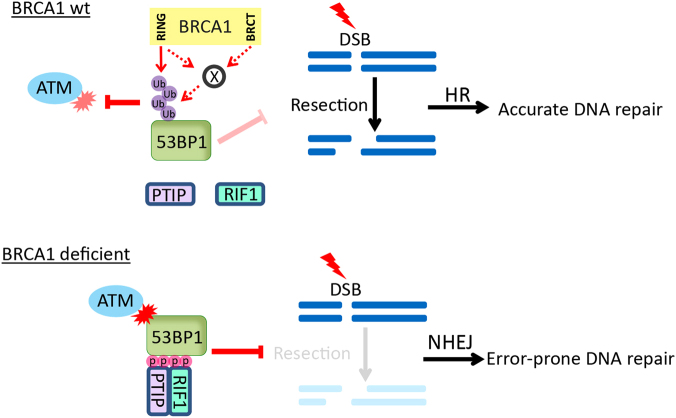
A working model for an inhibitory role of BRCA1 in the regulation of 53BP1 signaling.

Instead, we re-introduced several BRCA1 mutants to BRCA1-depleted cells and examined PTIP foci formation in cells synchronized in the S/G2 phases. Wild-type BRCA1 suppressed PTIP localization to DSBs, whereas the C61G mutant, as well as M1775R (a BRCT domain mutant that abolishes phospho-protein binding activity and DSB accumulation ability of BRCA1), behaved similar to loss-of-function mutants and failed to suppress PTIP foci formation in S/G2 cells (Figures 4d and e). The I26A mutant, which is a synthetic RING mutant that ablates the interaction of BRCA1 with all of the known cognate E2 enzymes *in vitro* [20, 21] only showed modest inhibition of PTIP foci formation when compared with the C61G mutant (Figures 4d and e). We speculate that the synthetic I26A mutant may be a hypomorphic mutant that still possesses residual activity *in vivo*. Alternatively, the intact RING domain, and/or the BRCT domain of BRCA1, may mediate the association of BRCA1 with another uncharacterized E3 ubiquitin ligase that is the bona fide E3 for 53BP1, or these domains may also mediate other protein–protein interactions that could prevent 53BP1 phosphorylation independent of its ubiquitination status. In summary, BRCA1 likely inhibits 53BP1 signaling through its RING and BRCT domains (Figure 5); however, the detailed mechanisms remain unclear.

## Discussion

Our findings reveal mechanistically how BRCA1 controls repair pathway choice and may provide a novel diagnosis marker for cancer patients with a *BRCA1* mutation. We demonstrate that BRCA1 restrains 53BP1 phosphorylation in the S/G2 phases to ensure the repair of DSBs by HR. On the other hand, 53BP1 inhibits HR and facilitates NHEJ by recruiting two downstream proteins to DSBs in an ATM-dependent manner [5–9, 11]. Previous studies on RIF1 have revealed the G1 phase-specific translocation of RIF1 to the DSB sites and the antagonizing role of BRCA1 for RIF1 foci formation in the S/G2 phases [5, 6, 10]. However, the mechanism by which BRCA1 competes with RIF1 and prevents RIF1’s localization to DSB sites in the S/G2 phases was unknown.

PTIP is another downstream effector of 53BP1 [9], but the foci formation of endogenous PTIP in different cell cycle phases had not been determined owing to a lack of antibodies that were suitable for detecting endogenous PTIP foci [9]. After creating a suitable PTIP antibody, we found that PTIP had the same cell cycle-dependent foci formation as RIF1, as revealed by both conventional microscope and laser-induced microirradiation (Figure 1 and Supplementary Figure S2), which prompted us to trace the upstream events that mediate the translocation of RIF1 and PTIP to the DSBs. Proper DSB localization and ATM-dependent phosphorylation of 53BP1 are required for RIF1 and PTIP foci formation. As 53BP1 forms foci in almost all cell cycle phases, we focused on ATM-mediated phosphorylation of 53BP1 in different cell cycle stages, and found that ATM-mediated 53BP1 phosphorylation occurs predominantly in G1 cells (Figure 2). These observations were contrary to a previous report, which did not detect cell cycle-dependent p-53BP1 foci formation [22]. The reason for this discrepancy is unknown. However, we showed here the cell cycle-dependent p-53BP1 in a large population of asynchronized cells (Figures 2a and b) and in synchronized cells (Figure 2d). Moreover, these data agree with the RIF1 and PTIP localization, which depends on 53BP1 phosphorylation. Strikingly, depletion of BRCA1 enables 53BP1 phosphorylation to occur in S/G2 phase cells, and subsequently, RIF1 and PTIP accumulate at the DSBs in the S/G2 phases (Figure 3) to inhibit DNA end processing and HR repair even in the presence of the homology template.

Mechanistically, BRCA1 does not attenuate ATM activation, as many ATM substrates are equally phosophorylated in cells with or without BRCA1. Unlike 53BP1, these ATM substrates did not show G1-specific phosphorylation (Figure 3c and Supplementary Figure S4). Moreover, the extent of DNA end resection is also not the determining factor for ATM-dependent 53BP1 phosphorylation, as CtIP depletion results in the same severe DNA end resection defects as does BRCA1 depletion, but fails to fully restore 53BP1 signaling in S/G2 cells (Supplementary Figures S5a and b). In addition, we also found that although depletion of 53BP1 largely restored CHK1 phosphorylation in response to camptothecin in BRCA1-deficient cells; however, the phosphorylation of RPA was not efficiently restored (Supplementary Figure S5c). CHK1 phosphorylation is a sensor for ssDNA–dsDNA junctions, whereas RPA phosphorylation levels reflect the length of ssDNA [23]. Together with previous studies on 53BP1/RIF and BRCA1, we propose that 53BP1 protects the double-stranded DNA from initial resection, which is mediated by BRCA1. However, once resection starts and the long stretches of ssDNA are being generated, 53BP1 complex can no longer protect them from processing. The reduced p-RPA in 53BP1/BRCA1 double-knockout (KO) cells may be due to additional functions of BRCA1 in DNA end resection. For example, a recent study indicates that the full activation of CtIP require its interaction with BRCA1 [24]. We hypothesize that BRCA1 may act both in early and late stages of DNA end resection, the former is achieved by antagonizing 53BP1, the latter may be mediated through CtIP interaction and activation. Of course, additional studies are needed to fully address this question.

53BP1 is present in BRCA1 complexes (Supplementary Figure S6a), a finding that is supported by previous report [25]. Moreover, we showed that BRCA1 ubiquitinates 53BP1, and that the C61G RING mutant fails to ubiquitinate 53BP1 efficiently. More important, reconstitution of the C61G mutant into BRCA1-deficient cells in the S/G2 phases was unable to suppress PTIP foci formation, implying that the E3 ligase activity of BRCA1 may be important for inhibiting 53BP1 phosphorylation. However, another BRCA1 RING mutant, I26A, seems to still preserve some ability to inhibit 53BP1, as both the percentage of PTIP foci-positive cells and the number of PTIP foci in each nucleus were lower in cells reconstituted with the I26A mutant than in cells reconstituted with the C61G mutant. Nevertheless, the extent of inhibition by the I26A mutant is milder than that of wild-type BRCA1 (Figure 4). These data support those of earlier studies that used the C61G and the I26A knock-in mice [26, 27]. It does not preclude the possibility that mutation of the Zn^2+^-chelating cysteine may affect local structural integrity of BRCA1 [22], which may not only just affect the E3 ligase activity of BRCA1, or the possibility that I26A still able to bind certain E2s, especially the E2s responsible for ubiquitin chain elongation [28].

We do not have enough evidence to support that BRCA1 directly ubiquitinates 53BP1 and prevents 53BP1 phosphorylation. *In vitro* ubiquitination assays showed that multiple regions of 53BP1 could be ubiquitinated by BRCA1 (Supplementary Figure S6a), which impede our attempts to map the ubiquitination sites on 53BP1 and further dissect the roles of BRCA1-mediated 53BP1 ubiquitination in DNA repair choice and the prevention of 53BP1 phosphorylation and functions. Thus, a direct role of BRCA1-dependent ubiquitination events in preventing 53BP1 phosphorylation remains unknown. It is possible that even though BRCA1 could ubiquitinate 53BP1, its ability to inhibit 53BP1 may be mediated by other BRCA1-dependent events, which require both its RING and BRCT domains.

We propose that in normal S/G2 cells, BRCA1 may directly or indirectly preclude or reduce its phosphorylation by ATM, although the detailed mechanisms remain unknown. It is possible that the inhibition of 53BP1 phosphorylation subsequently prevent the recruitment of RIF1 and PTIP to DSBs. The broken DNA ends are therefore processed by nucleases involved in resection. Once the long ssDNA regions are generated, these broken DNA are destined for repair by the high-fidelity HR process. However, in breast and ovarian cancer cells with BRCA1 deficiency, 53BP1 signaling in the S/G2 phases is no longer inhibited, and RIF1 and PTIP translocate to DNA break sites via binding to phosphorylated 53BP1 to protect DNA ends from processing. As a result, HR is suppressed and the error-prone NHEJ takes over (Figure 5), which may lead to the accumulation of chromosome aberrations and eventually lead to tumorigenesis.

The most promising strategy for treating HR-deficient tumors, such as BRCA1-mutated breast and ovarian cancers, is the use of PARP inhibitors [29]. Although a significant percentage of *BRCA1* mutation carriers responded to poly(ADP-ribose) polymerase inhibitors (PARPi), some patients were resistant to this treatment. Breast cancers arising in BRCA1 mutation carriers frequently showed low levels of 53BP1 expression. Interestingly, a fraction of PARPi-resistant tumors restored HR yet did not lose 53BP1 [30]. We envision that 53BP1 hypo-phosphorylation and ATM inactivation, along with the failure of RIF1 and PTIP foci formation, may emerge as novel indicators for PARPi resistance in BRCA1-mutated cancer patients.

## Materials and Methods

### Antibodies

The following antibodies were used in this study: anti-FLAG (F3165, Sigma, St Louis, MO, USA); anti-BRCA1 (D-9, Santa Cruz, Dallas, TX, USA); anti-cyclin A (sc-751 and sc-271645, Santa Cruz); anti-NBS1 (A301-289A, Bethyl, Montgomery, TX, USA); anti-53BP1(612522, BD, San Jose, CA, USA); anti-phospho-ATM (S1981) (4526, Cell Signaling, Danvers, MA, USA); anti-phospho-RPA32 (S4/S8) (A300-245, Bethyl); anti-phospho-53BP1 (S25/29) (2674, Cell Signaling); anti-phospho-53BP1 (S1778) (2675, Cell Signaling); anti-phospho-NBS1 (S343) (3001, Cell Signaling); anti-phospho-SMC1 (S957) (4801, Cell Signaling) anti-phospho-CHK1 (S345) (2348, Cell Signaling); anti-phospho-KAP-1 (S824) (4127, Cell Signaling) and anti-phospho-(Ser/Thr) ATM/ATR substrate antibody (2851, Cell Signaling). Rabbit anti-PTIP antisera were obtained from rabbits immunized with GST-PTIP (residues 590–1 069) fusion protein expressed and purified from *Escherichia coli*. Antisera were affinity-purified using AminoLink Plus Immobilization and Purification Kit (Pierce, Waltham, MA, USA ). Rabbit polyclonal antibodies against BRCA1, RIF1, ATM, γH2AX, 53BP1 and phospho-CHK2 (T68) were described previously [5, 31–33].

### Cell cycle synchronization

Synchronization methods were similar to those described previously [5]. Cells were released from double-thymidine block for 6 h to synchronize in S/G2 phases.

### Cell culture, cell transfection, short hairpin RNAs and fluorescence microscopy

The methods used to culture human cells, the sequences of short hairpin RNAs, microscopy and laser-induced microirradiation methods were described previously [5, 31].

### Tandem affinity purification, immunoprecipitation, *in vivo* and *in vitro* ubiquitination asssay

Tandem affinity purification were performed as described previously [31]. As for immunoprecipitation assay, cells were irradiated (10 Gy) or untreated and harvested 1 h later. Cells were lysed in NETN buffer (20 mM Tris-HCl at pH 7.0, 100 mM NaCl, 1 mM EDTA and 0.5% Nonidet P-40) containing 50 mM β-glycerophosphate, 10 mM NaF and 1 μg ml^−1^ each of pepstatin A and aprotinin, and 5 mM N-Ethylmaleimide (NEM). After centrifugation, the supernatant (soluble fraction) were collected and the pellet was sonicated in high-salt solution (20 mM Tris-Hcl at pH 7.0, 0.5 M NaCl, 1 mM EDTA, 1 mM EGTA, protease and phosphatase inhibitor and 5 mM NEM) to extract chromatin-bound protein fractions. The supernatants were combined and centrifuged again at 14 000 r.p.m. to remove debris and then subjected to immunoprecipitation. For ubiquitination assay, the total lysates were immunoprecipitated with normal rabbit immunoglobulin G or anti-53BP1 polyclonal antibodies for 4–6 h at 4 °C. This was followed by incubating the mixture with protein A/G agarose for an additional hour. Immunoprecipitates were washed three times with high-salt NETN buffer (20 mM Tris-HCl at pH 7.0, 500 mM NaCl, 1 mM EDTA and 0.5% Nonidet P-40) and once with normal NETN buffer to dissociate non-covalently bound ubiquitin. Nickel-nitrilotriacetic acid pull-down under denaturing condition was performed as described previously [34]. GST-53BP1 fragments and His-BRCA1_1-304_/His-BARD1_26-327_ dimer were purified from *E. coli* and co-incubated for 1 h at 30 °C for *in vitro* ubiquitination assays, as described previously [20, 32].

### ATM kinase assay

ATM was immunoprecipitated from 293T cells with anti-ATM antibody, and the kinase assay was performed as previously described [33]. GST-53BP1 (residues 1–910 aa) fusion protein was purified from *E. coli* and used as a substrate for ATM.
