# Supplementary material for: Cell cycle-dependent inhibition of 53BP1 signaling by BRCA1
Source: Cell Discov. 2015 Aug 4;1:15019–. doi: 10.1038/celldisc.2015.19 (PMC4860855; doi:10.1038/celldisc.2015.19)
Supplement: Supplementary Information [file celldisc201519-s1.doc]

**Supplementary information**

**CRISPR/Cas9-based approach to generate 53BP1, PTIP, BRCA1 single knockout and BRCA1/53BP1 double knockout cells**

Vectors encoding guide RNA (gRNA) were digested with AfIII, and a pair of annealed oligonucleotides for each targeting site was cloned into the gRNA vector according to the protocol . Designs were chosen to target the gene in one of the first few exons and were tested for obvious potential off-targets by bioinformatics analysis. 293T or HeLa cells were co-transfected with gRNA-expression vector and the hCas9 vector. Cells were seeded as single colonies in 96-well plates. After 2–3 weeks, clones were selected based on Western blotting and immunofluorescence analysis. The gRNA target sequences are: 53BP1: GAAAGTTCGGCTTACCTTGC and PTIP: GTGTGAGGCTAGTGCATTGT.

**References**:

Cong, L., Ran, F.A., Cox, D., Lin, S., Barretto, R., Habib, N., Hsu, P.D., Wu, X., Jiang, W., Marraffini, L.*A., et a*l. (2013). Multiplex genome engineering using CRISPR/Cas systems. Scien*ce 3*39, 819-823.

**Supplementary Figure Legends**

**Fig. S1**. Validation of PTIP antibody. This supplemental figure is related to **Figure 1**. (**a**) PTIP antibody validation by Western blotting. Control 293T cells and 293T PTIP knockout cells were harvested and subjected to Western blot analysis using antibodies against PITP and tubulin. Molecular weights (kDa) were indicated. (**b**) PTIP localization in undamaged cells was shown in the upper panel. IR-induced PTIP localization in 293T cells, 293T cells treated with ATM inhibitor KU55933 (10 μm), 293T PTIP knockout cells, or 293T 53BP1 knockout cells were shown in the lower panel. (**c**) IR-induced PTIP localization in MCF10A cells.

**Fig. 2.** Recruitment of PTIP and BRCA1 to DSBs generated by laser-induced microirradiation. U2OS or MCF10A cells were irradiated with a 364 nm laser line. Cells were recovered for 20 min before fixation. Cells in different phases of cell cycle were indicated.

**Fig. S3.** Validation of p-53BP1 antibodies. This supplementary figure is related to **Fig. 2**. (**a**) 53BP1 and p-53BP1 staining in undamaged cells were shown in the upper panel. Immunostaining using anti-53BP1 antibody and anti-phospho-53BP1 (S25/29) or anti-phospho-53BP1 (S1778) antibodies in irradiated normal and 53BP1 knockout cells were shown in the bottom panel. (**b**) Phosphorylation on S25/29 and S1778 sites were ATM-dependent. HeLa cells were pretreated with DMSO, ATM inhibitor KU55933 (10 μm), or DNA-PK inhibitor NU7026 (2.5 μm) and then irradiated (10 Gy) or untreated. 1 h later cells were harvested and the lysates were analyzed by Western blotting using indicated antibodies.

**Fig. S4**. Immunofluorence staining for some ATM substrates after IR. This supplementary figure is related to **Fig. 2a-b**. Anti-p-S/TQ is an antibody that recognizes phosphorylated ATM/ATR consensus sites.

**Fig. S5**. CtIP depletion is unable to rescue 53BP1 signaling. This supplementary figure is elated to **Fig. 3a-b**. (**a**) HeLa cells infected lentivirus carrying CtIP shRNA were synchronized S/G2 phase and processed as described in Figure 3A, the percentage of cells forming discrete p-53BP1 and PTIP foci were quantified. (**b**)DNA-end resection defects in BRCA1 and CtIP-depleted cells. Control, BRCA1 or CtIP-depleted cells were either left untreated or treated with 0.5 μM camptothecin (CPT) for the indicated time. CPT is a DNA topoisomerase I inhibitor that induce robust activation of ATR signaling. The extent of RPA phosphorylation depends on the length of ssDNA generated by DNA-end resection. (**c**) DNA-end resection defects in BRCA1 and 53BP1-co-depleted cells. Control or 53BP1-knockout HeLa cells were transfected with control or BRCA1 siRNAs. 48 h later the cells were treated and processed as in **b**.

**Fig. S6**. Identification of 53BP1 as a component in BRCA1/BARD1 complex. This supplementary figure is related to **Fig. 4**. (**a**) 293T cells stably expressing SFB-tagged (S-tag, Flag epitope tag, and streptavidin-binding peptide tag)-BRCA1 or BARD1 were used for Tandem Affinity Purification (TAP). Tables are summaries of proteins identified by Mass spectrometry analysis. (**b**) *In vitro* ubiquitination of 53BP1 by BRCA1. Recombinant GST-53BP1 fragments including N-terminus (1-1051aa), C1 (1052-1300aa), C2 (1301-1639aa), and C3 (1640-1972aa) regions were incubated with E1, E2 (UbcH5a), ubiquitin, and ATP in the absence or presence His-BRCA1_1-304_ /His-BARD1_26-327_ dimer. After 1 h at 30°C, the reactions were stopped by the addition of SDS-loading buffer and analyzed by SDS-PAGE. Immunoblotting was conducted using anti-GST antibody. The lower-mobility bands corresponded to mono, oligo, or poly-ubiquitinated 53BP1 fragments.
