# Supplementary figures and images for: Cell cycle-dependent inhibition of 53BP1 signaling by BRCA1
Source: Cell Discov. 2015 Aug 4;1:15019–. doi: 10.1038/celldisc.2015.19 (PMC4860855; doi:10.1038/celldisc.2015.19)

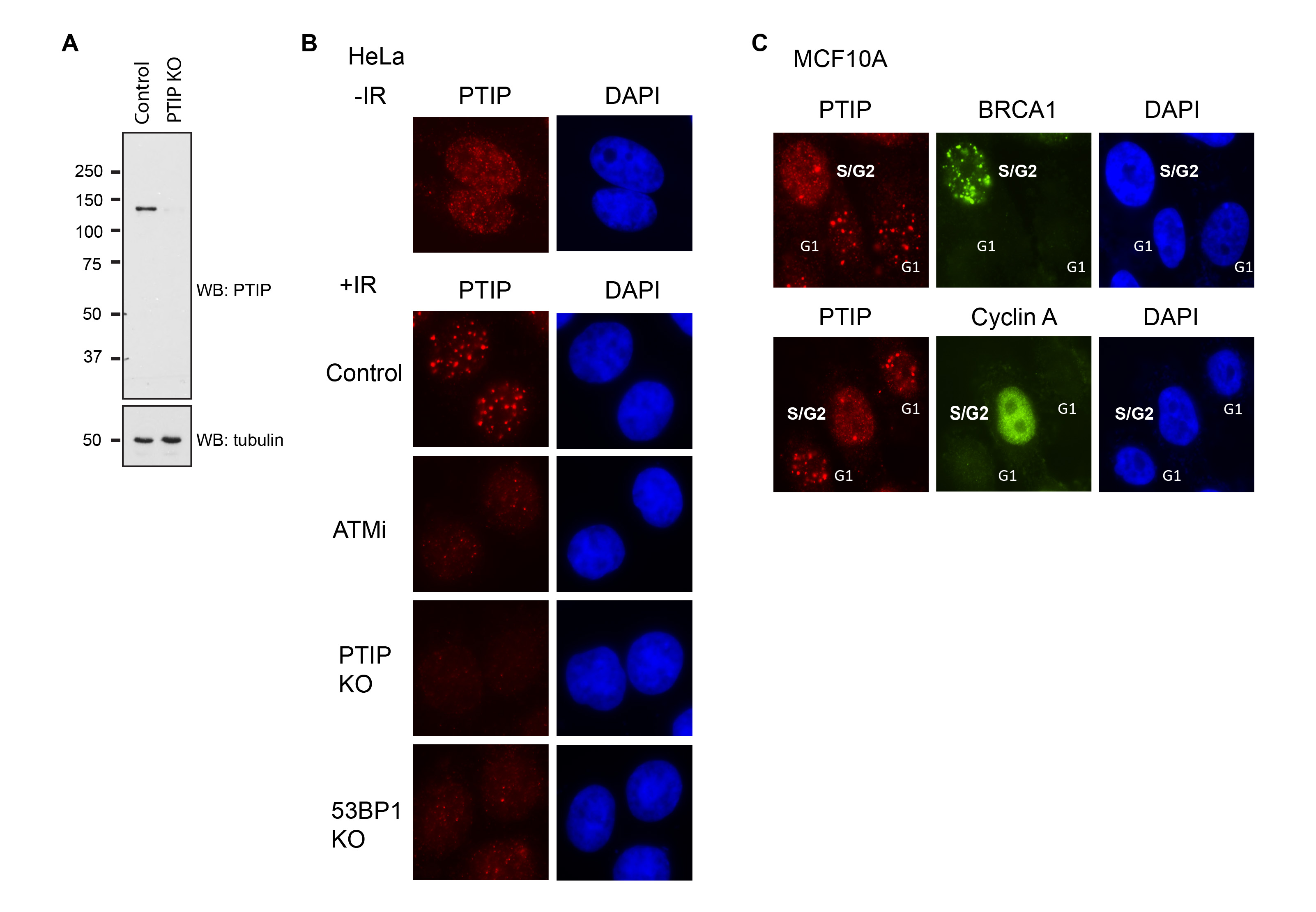

Supplement: Supplementary Figure S1 [file celldisc201519-s2.jpg]

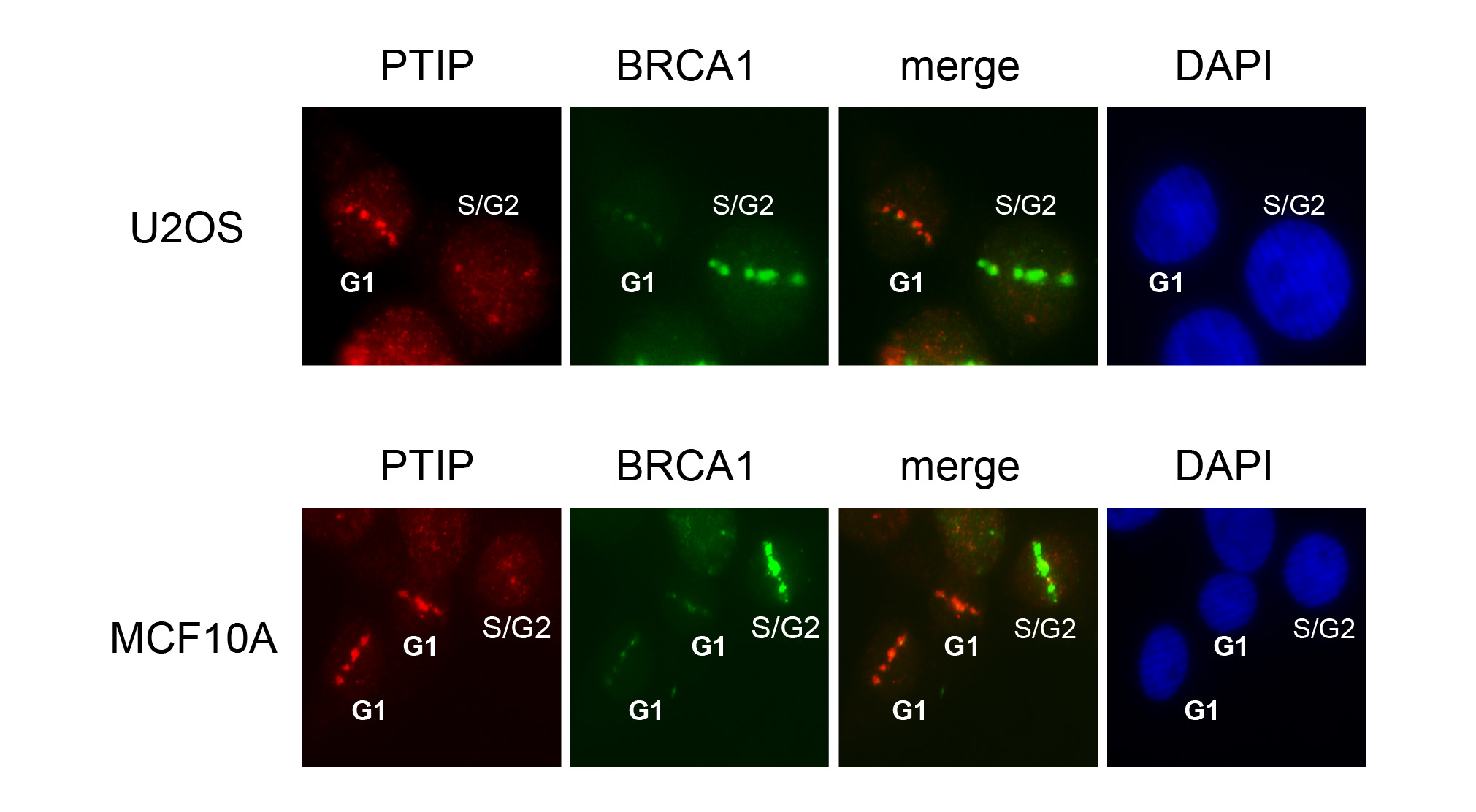

Supplement: Supplementary Figure S2 [file celldisc201519-s3.jpg]

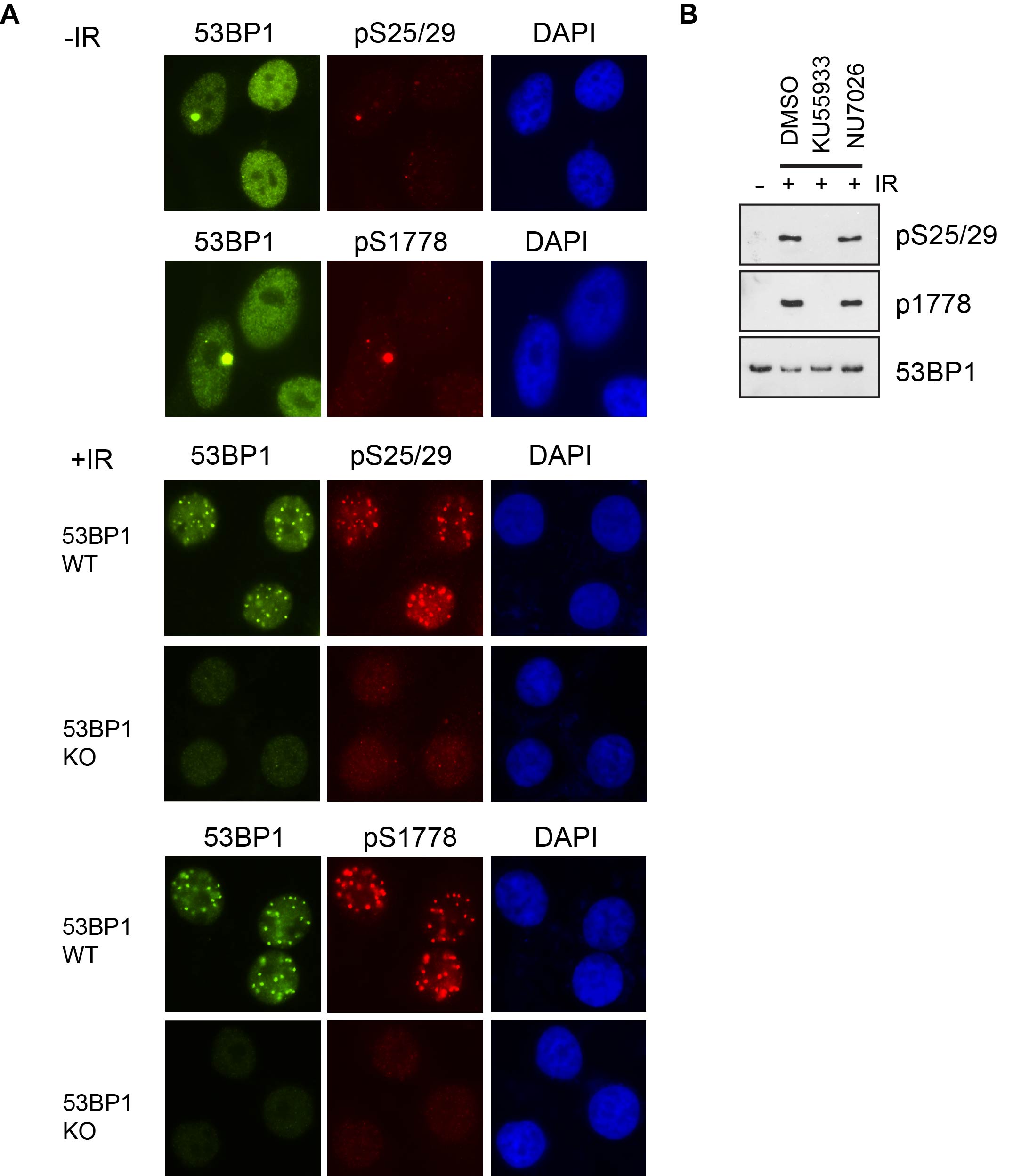

Supplement: Supplementary Figure S3 [file celldisc201519-s4.jpg]

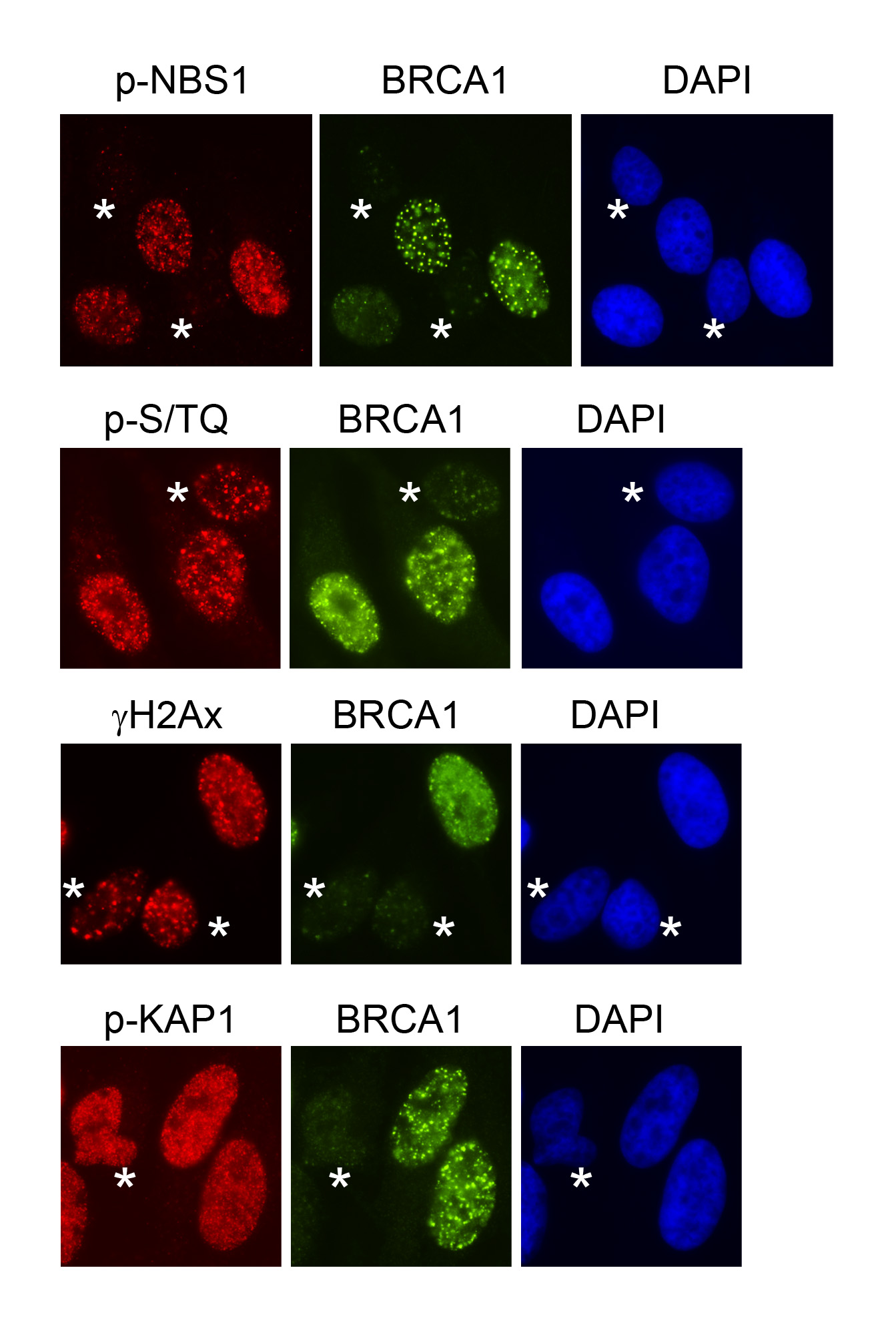

Supplement: Supplementary Figure S4 [file celldisc201519-s5.jpg]

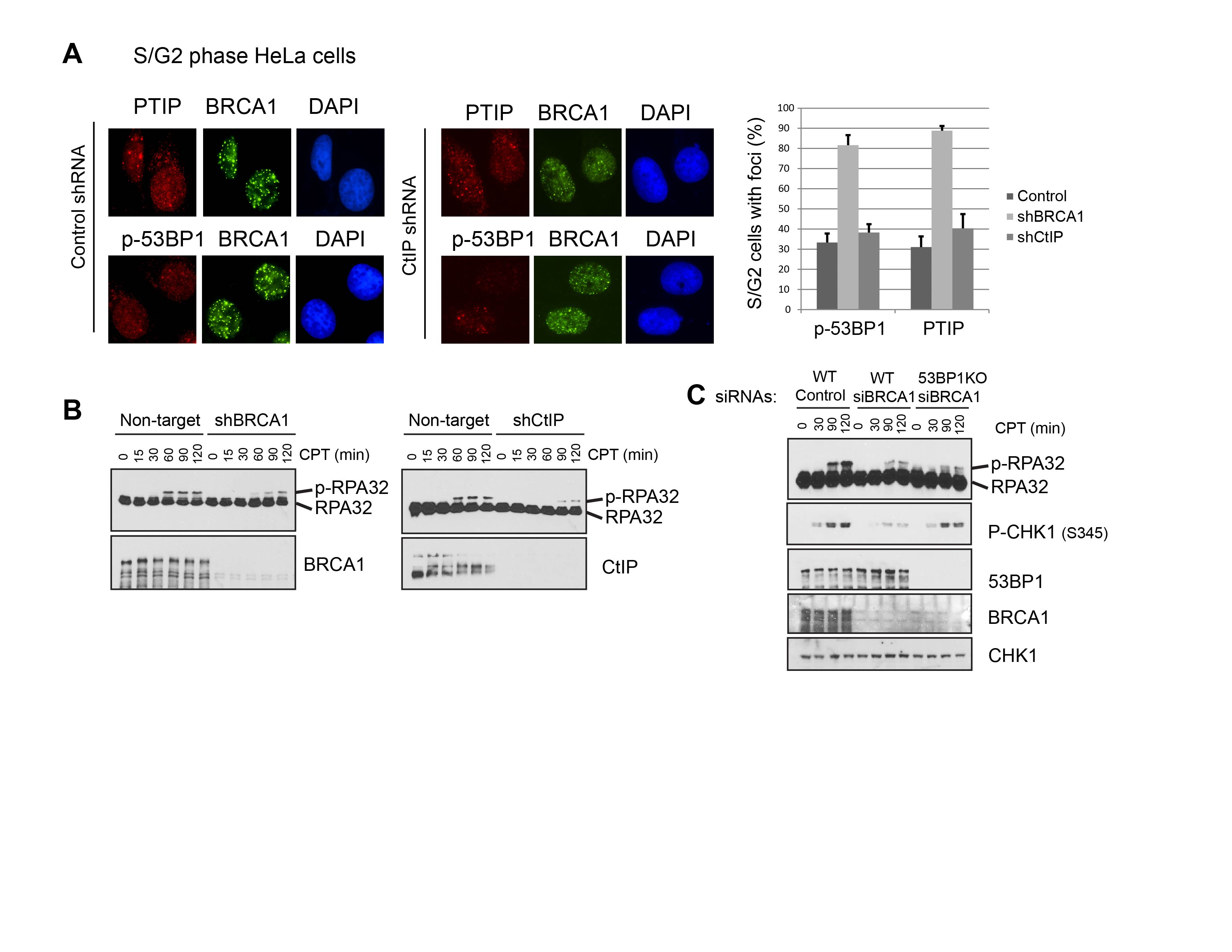

Supplement: Supplementary Figure S5 [file celldisc201519-s6.jpg]

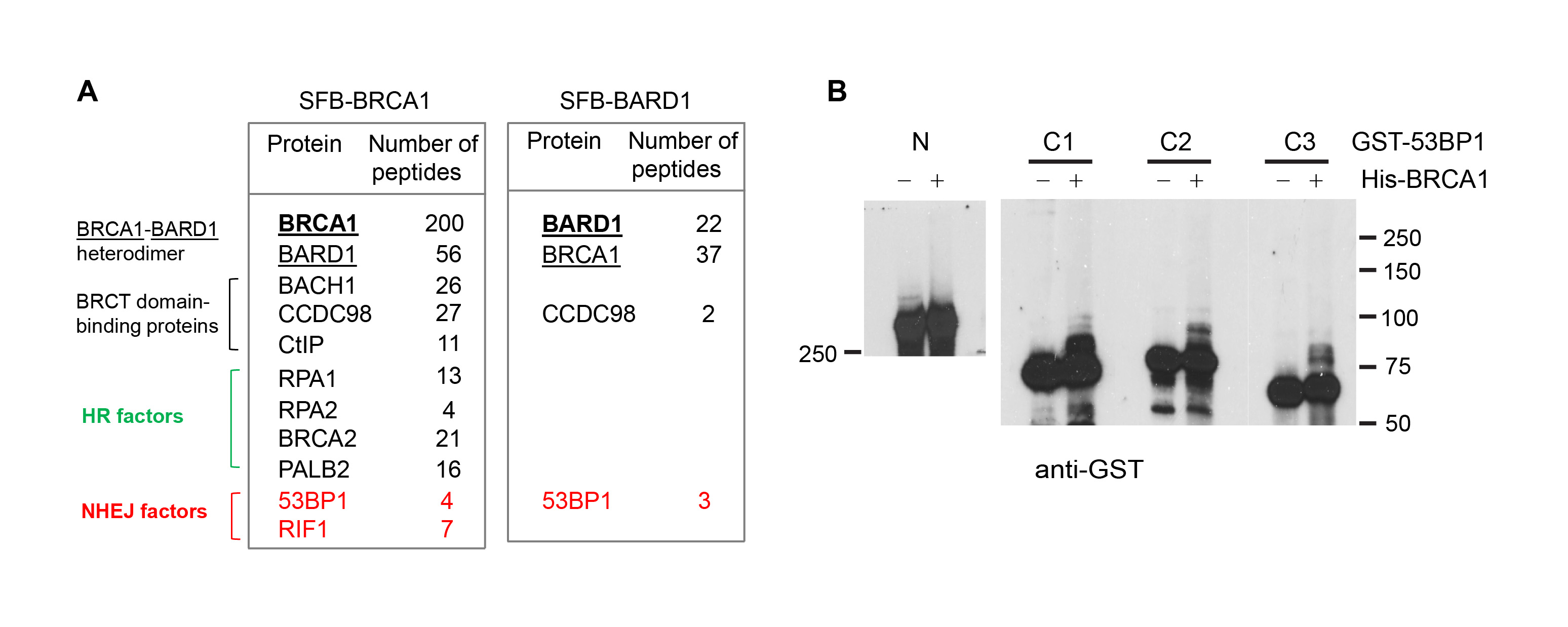

Supplement: Supplementary Figure S6 [file celldisc201519-s7.jpg]
